# Why 'piss' is ruder than 'pee'? The role of sound in affective meaning making

**DOI:** 10.1371/journal.pone.0198430

**Published:** 2018-06-06

**Authors:** Arash Aryani, Markus Conrad, David Schmidtke, Arthur Jacobs

**Affiliations:** 1 Department of Experimental and Neurocognitive Psychology, Freie Universität Berlin, Berlin, Germany; 2 Department of Cognitive, Social and Organizational Psychology, University of La Laguna, La Laguna, Spain; 3 Centre for Cognitive Neuroscience Berlin (CCNB), Berlin, Germany; Kyoto University, JAPAN

## Abstract

Most language users agree that some words sound harsh (e.g. grotesque) whereas others sound soft and pleasing (e.g. lagoon). While this prominent feature of human language has always been creatively deployed in art and poetry, it is still largely unknown whether the sound of a word in itself makes any contribution to the word’s meaning as perceived and interpreted by the listener. In a large-scale lexicon analysis, we focused on the affective substrates of words’ meaning (i.e. *affective meaning*) and words’ sound (i.e. *affective sound*); both being measured on a two-dimensional space of valence (ranging from pleasant to unpleasant) and arousal (ranging from calm to excited). We tested the hypothesis that the sound of a word possesses affective iconic characteristics that can implicitly influence listeners when evaluating the affective meaning of that word. The results show that a significant portion of the variance in affective meaning ratings of printed words depends on a number of spectral and temporal acoustic features extracted from these words after converting them to their spoken form (study1). In order to test the affective nature of this effect, we independently assessed the affective sound of these words using two different methods: through direct rating (study2a), and through acoustic models that we implemented based on pseudoword materials (study2b). In line with our hypothesis, the estimated contribution of words’ sound to ratings of words’ affective meaning was indeed associated with the affective sound of these words; with a stronger effect for arousal than for valence. Further analyses revealed crucial phonetic features potentially causing the effect of sound on meaning: For instance, words with short vowels, voiceless consonants, and hissing sibilants (as in ‘piss’) feel more arousing and negative. Our findings suggest that the process of meaning making is not solely determined by arbitrary mappings between formal aspects of words and concepts they refer to. Rather, even in silent reading, words’ acoustic profiles provide affective perceptual cues that language users may implicitly use to construct words’ overall meaning.

## Introduction

Human language has generally been considered to be entirely symbolic in that words convey meaning through conventional and arbitrary links to concepts they refer to [1]. From this perspective, phonemes (i.e. the speech sounds that constitute words) have no inherent semantic content nor have they any stand-alone contribution to words’ meaning. Nevertheless, even a naïve reader—without prior knowledge of such literary devices as cacophony or euphony—would experience how, for instance, in Poe’s verse “…*Hear the loud alarum bells—Brazen bells*!*—What tale of terror*, *now*, *their turbulency tells*!” [2], the explosive consonant /t/ and other harsh and discordant sounds (e.g. hissing sibilants /s/ and /z/) evoke a feeling of “terror” provoked by “brazen” bells.

Within literary studies, many have noted that poetry achieves much of its affective aesthetic impact through sound manipulation, and that phonological structure has a semantic function beyond the decorative [3–5]. In a similar fashion, swear words usually possess specific phonological patterns that can potentially amplify the negative emotional response that they mean to evoke [6]. Looking at the famous seven words listed by American comedian George Carlin that “you can never say on television” [7] reveals that all of these words contain voiceless stops (/t/ and /k/) or hissing sibilants (/s/ and /ʃ/), which are fortis consonants, articulated with greater oral pressure and relatively higher muscular force compared to their lenis counterparts.

However, despite the fact that influential linguists and experimental psychologists throughout the last century promoted the idea that the sound of a word may have a synchronic, productive effect on overall meaning construction [8–10], the notion of the arbitrariness of the linguistic sign [1] has generally dominated research on human language.

More recently, a growing body of research challenges the idea of absolute arbitrariness by providing evidence for non-arbitrary sound-to-meaning correspondences (see [11–13] for reviews) including some universal patterns across various languages of the world [14]. These results assign a supplementary function to sound-to-meaning correspondences that structure vocabulary [15,16] and play an important role for both phylogenetic language evolution [16–18] and ontogenetic language development [18,19]. Nonetheless, despite the increasing number of studies examining sound-to-meaning associations, to the best of our knowledge, there has been no empirical study examining whether specific properties in the sound of a real word play a part in contributing to its overall meaning. With the present study, we aimed at addressing this research question. By focusing on the ‘affective meaning’ of words, and by providing reliable quantitative measures for ‘affective sound’ of words, we investigated how the sound of a word potentially contributes to its meaning as perceived and evaluated by the listener. A further goal of this study was to explore the affective acoustic cues and their underlying phonetic features that may implicitly influence language users when evaluating words’ affective meaning.

### Motivation for the present study

Our approach was motivated by a number of limitations evident in previous work. Experimental research based on behavioral data has hitherto merely investigated the links between some selective, rather isolated attributes of meaning (e.g. the physical size of the referent) and some aspects of sound (e.g. intrinsic pitch of vowels) mainly by using nonword stimuli (see [20] for supporting a graded relationship between sound and meaning, and [21], for an evolutionary perspective on the phenomenon). Such approaches exhibit three major limitations that we aimed to address in the present study.

The first limitation relates to the focus on semantic effects of phonemes in nonwords instead of natural words. Such studies are motivated by the fact that natural words in a language are linked to predetermined semantic concepts that are automatically activated during word recognition. In order to disentangle the effect of phonology from that of semantics, the majority of previous studies therefore relied on nonword stimuli usually used in a forced-choice paradigm thus limiting the generalizability of the results to real words. For instance, the phonemes /ɑ/ and /ɪ/ when used in experimentally manipulated nonwords—as in “mal” and “mil” in the seminal study by Sapir [9]—have repeatedly been suggested to denote big and small objects, respectively [11,12]. However, in a natural language like English, they appear in the corresponding semantic concepts in the opposite way: /smɑl/ and /bɪɡ/. This begs the question to what extent the results of these studies can be linked to natural language processing and whether the assumed quality of phonemes has, if any, effects on the evaluation of meaning for real words.

A second issue relates to the problem of deciphering the likely cause(s) of sound-to-meaning correspondences. Proposals on non-arbitrariness of language distinguish between two types of motivations for such sound-meaning mappings [12]: Iconicity, which is based on perceptual similarities between sound and meaning (e.g. onomatopoeia), versus systematicity which is based on statistical regularities in language that link specific patterns of sound to specific semantic or grammatical concepts [22,23]. Besides some familiar and straightforward examples of iconicity—such as onomatopoetic words—research in this field still faces the question of whether existing findings on the relationship between sound and meaning are caused by specific distributions of phonemes in a language (i.e. systematicity), or by perceptual qualities that phonemes inherently convey (i.e. iconicity). The phonaestheme /sn-/ appearing as an initial sound cluster in many English words related to ‘mouth’ or ‘nose’ may serve to illustrate this dilemma [24]. In this case, there has been no empirical support showing whether there is a specific (nasal) quality in the sound of /sn-/ that is linked with the concepts of ‘mouth’ or ‘nose’, or rather the organization of the vocabulary is designed in a way that this specific sound cluster over-proportionally appears in words that are related to these concepts.

The third and presumably most important issue is that the operationalization of meaning in this field of research has so far been restricted to only some selective aspects of sensorimotor information (e.g. shape, movement). The role of affect as a most basic human experience shaping the learning, representation, and processing of language [25–29] has been surprisingly neglected. Indeed, affective dimensions of words, in particular, valence and arousal, are essential features defining a two-dimensional semantic space allowing for a very basic and potentially the most relevant distinction between different concepts; as empirically established by semantic differential [30]. In an attempt to provide a quantitative measure for words’ meaning, Osgood [30] defined 100 different lexical dimensions and asked participants to allocate the meaning of words for each dimension in an experiential continuum definable by a pair of polar terms (e.g. soft/hard, long/short, angular/rounded). Factor analyses conducted on the wide variety of verbal judgments indicated that most of the variance was accounted for by three major semantic dimensions: The two primary dimensions of ‘valence’ and ‘arousal’, and a third, less strongly-related dimension (in terms of the explained variance) of ‘dominance’ or ‘control’ [31]. Therefore, these factors have been considered basic dimensions of the semantic space within which the meaning of any concept can be specified.

Moreover, the expression and perception of affective states are fundamental aspects of human communication [32,33] that have been proposed as the original impetus for language evolution; with mimetic vocalization of emotional sounds supposedly allowing early hominids to efficiently share biologically significant information [32,34,35]. Therefore, we would expect the effect of iconicity to be most evident in the communication of affect and in the relationship between words’ affective sound (i.e. how emotionally words sound) and words’ affective meaning (i.e. their position in the bi-dimensional affective space of lexical valence and arousal). Thus iconicity can serve as an interface for accomplishing the need to map linguistic form to human affective experience as a vital part of meaning making.

### An embodied view on affective meaning

It is important to consider that the notion of “affective meaning” may not be shared by all theories on linguistic meaning. Our approach in this work is based on an embodied view of language which proposes that meaning is grounded in behavior (perception and action) and neural circuitry of the producer or the interpreter of linguistic signs [25,28,36–40]. Ultimately, part of the meaning of any utterance is its effect on the (physical and emotional) well-being of the person saying or hearing it, and everything that matters is represented in each individual person’s brain and its neurophysiological systems. Presumably, the most fundamental such system is affect: in order to make meaning, we need to know what object/event in our environment requires us to react with alert or to keep calm, to approach or to withdraw. Moreover, the ability to distinguish between such affective contexts or reactions is linked to attention systems that select specific sensory input for further processing, and also to motor systems that select specific actions for output. Both systems (i.e. sensory and motor) provide crucial information for the construction of meaning by language users. Findings on the role of affective meaning in modulating various cognitive processes, such as learning, memory, attention or language processing, [25,26,28,41] support the idea that affective meaning is intertwined with other lexico-semantic aspects and has an essential and basic contribution to the process of meaning making.

### The present study

We addressed the above-mentioned problems apparent in previous research by focusing on the affective meaning of real words and investigated whether participants were implicitly influenced by words’ sound while giving a rating on emotion expressed in words’ meaning.

Specifically, we aimed to challenge the established notion that assigning (affective) meaning to words is merely determined by words’ semantic content and by an associative and per se arbitrary relationship between the signifier (sound image) and the signified (concept)–as encouraged by a leading principle of modern linguistics [1]. Instead, we propose that the overall affective meaning of a word is, in addition to the word’s semantic content, co-determined by inherent qualities of the signifier and by the percept derived from words’ acoustic-phonetic features (i.e. the *affective sound*). Note that our use of the term affective sound in this paper refers exclusively to phonological constituents of words themselves and not to speaker-related issues such as intonation or how a word is spoken.

Our main hypothesis is motivated by research on nonverbal emotional vocalization and, in particular, emotional prosody which has shown that the emotional significance of a sound can be detected, and hence be integrated with higher-order cognition, even when the attentional focus is not directly on the emotional cues of the sound [42–45]. Such emotional cues have been shown to be engaged even in silent reading by means of cross-sensory input from the visual cortex into the auditory cortex and affective regions in the brain [46,47]; as put forward by theories of embodied cognition and perceptual simulation. On the other hand, phonemes and their combinations (as in words) are characterized by a number of acoustic features that overlap with those that modulate emotional vocalization and emotional prosody (e.g. sound formants, sound intensity). Therefore, the specific sound profile of any word in a language can theoretically be attributed to a specific emotion as perceived by the listener. We thus hypothesized that the process of affective lexical evaluation—as higher order cognition—is influenced by words’ phonology: that is, their phonologically recoded neuronal representation of the acoustic features corresponding to phonological word forms [48–51].

We used a large-scale normative database including rating values for affective meaning of words that were evaluated by at least 20 subjects/item. In line with Osgood’s semantic differential [30], such databases usually contain two types of ratings. The first component concerns emotional valence going from unpleasant to pleasant. For instance, words such as “murder”, “poison”, and “virus” are commonly rated as “very unpleasant” whereas “freedom”, “love”, and “life” lie on the other extreme end. The second type of ratings addresses the degree of emotional arousal ranging from excited (e.g. “nightmare”, “sex”, and “courage”), to calm (e.g. “health”, “massage”, and “peace”). Using these ratings as a measure of words’ affective meaning, we tested the null hypothesis (H0) that explicit evaluations of affective meaning solely reflect written words’ semantic content, against the alternative hypothesis (H1) that phonological word forms also contribute to valence and arousal ratings so that a statistically significant portion of their variance can be accounted for by words’ acoustic features. For instance, harsh-sounding words might make people feel more aroused so that they implicitly give a higher arousal rating, even though they are instructed to only focus on the lexico-semantic aspect of words.

In order to test H1, we chose a computational approach that employs signal averaging to amplify the potential effect of sound on meaning. Subsequently, we quantified a *Phonological Affective Potential* (PAP) of words, separately for arousal (PAP_aro_) and for valence (PAP_val_), both estimates representing the influence of words’ affective sound on their affective meaning. The goal of this work is to examine the psychological reality of the PAP, and to test whether the PAP is linked to (and derived from) words’ acoustic features that we extracted from their spoken forms after synthesizing them (study 1). Note that according to H0 the amount of variance in the PAP that depends on words’ acoustic features should not be greater than chance level and therefore statistically not significant. Next, we tested the association of the PAP with words’ affective sound assessed in two behavioral studies using different methods. We first asked participants to rate the affective sound of printed words while suppressing words’ meaning (study 2a), and second, we employed auditory presented pseudowords and acoustic models to predict words’ affective sound based on their acoustic features (study 2b). We then compared the PAP with these two independent measures of affective sound to test for their potential associations. Finally, in order to identify the perceptual cues potentially underlying the effect of implicit sound on meaning, we separately tested the relationship between words’ acoustic features and words’ PAP and the two independent measures for words’ affective sound.

Although previous studies provided first affirmative support for the affective potential of some phonological units [52–56], ours is the first study demonstrating the psychological reality of phonemes’ affective potential and the contribution of words’ implicit sound to meaning making for real words and across a language’s lexicon.

## Quantifying the *Phonological Affective Potential* (PAP)

### Material

The Berlin Affective Word List [57] (BAWL) was used as a normative database containing a representative mass of 2694 German words that has been cross-validated in various empirical studies regarding experiential, behavioral, and neurobiological levels of analysis [40]. The BAWL includes words from different classes (nouns, verbs, and adjectives) that were selected based on the following main criteria: to include a) the most frequently used German words, b) as many words as possible with an apparent relation to affect regardless of whether this would result in more or less extreme values of valence and arousal, and c) a critical mass of theoretically neutral words. As a consequence, the BAWL contains a relatively elevated percentage of emotion-laden words. However, valence and arousal values of words in the BAWL are spread across the entire range of both valence and arousal in order to make it an optimal tool for selecting verbal material for all kinds of research questions on affective language processing. Henceforth, we refer to these valence and arousal values representing words’ affective meaning as *Affective Meaning Ratings*.

### Method

We operationalized H1 by assigning two statistical components to each of the *Affective Meaning Ratings* in the BAWL (separately for arousal and valence): for a word composed of phonemes *W*_*i*_ [*ph*_*1*_, *ph*_*2*_*…*, *ph*_*n*_], the rating value was considered to reflect a first component stemming from an explicit evaluation of the word’s *Affective Semantic Content*, and a second phonological component, the PAP (*Phonological Affective Potential*), which reflects the contribution of the affective potential of phonemes to the total rating value. Assuming a simple additive model, the rating for a given word in the database can thus be modelled as the sum of these two components plus an error term ϵ

$$AffectiveMeaning\;Rating\left(W_{i}\right)=Affective\;Semantic\;Content\left(W_{i}\right)+PAP_{i}\left[ph_{1},\;ph_{2},\ldots ,\;ph_{n}\right]+\varepsilon$$ (1)

We estimated the PAP by averaging the potential affective effects of all phonemes regardless of their position in the word. The PAP thus can be hypothesized as a function of the *Phonological Affective Value* (PAV) of each phoneme: that is, the contribution of the affective quality of each single phoneme to the total rating value for each word:

$$PAP_{i}\left[ph_{1},\;ph_{2},\ldots ,\;ph_{n}\right]=Mean\;\left[PAV\left(ph_{1}\right),\;PAV\left(ph_{2}\right),\ldots ,\;PAV\left(ph_{n}\right)\right]$$ (2)

**Fig 1:**
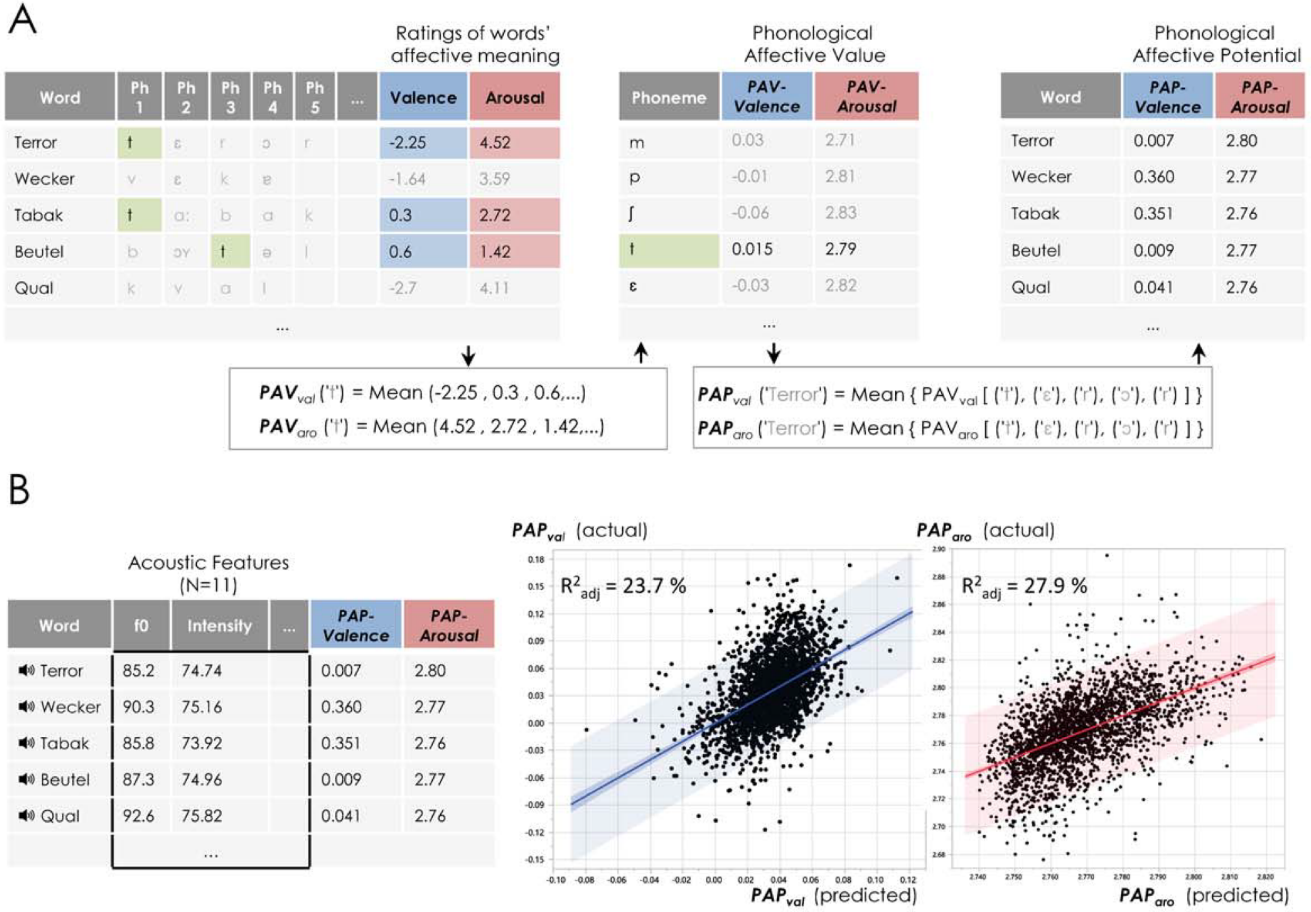
**A)** Words in the normative database (BAWL) were segmented and coded for the presence or absence of a given phoneme (here exemplified by the phoneme /t/). The phonemes were analyzed one-by-one to determine their potential effect on valence and arousal ratings. The potential affective effect caused by each single phoneme (i.e. PAV) was computed as the average of valence or arousal ratings of words containing this specific phoneme. The PAP of each word was calculated as the average of all its PAVs. **B)** Words were synthesized and their extracted acoustic features were used in two multiple linear regression models as predictors for the PAP of arousal (right) and valence (left). The acoustic variables (11 in total) accounted for 27.9% and 23.7% of the variance in PAP_aro_ and PAP_val_ respectively (study 1).

In order to quantify the PAV(*ph*_*j*_), we considered it to be a signal masked by ‘noise’: that is, the *Affective Semantic Content* (*W*_*i*_) and the error term in Eq 1. We thus attempted to minimize the effect of noise while increasing the signal-to-noise-ratio (SNR) by averaging. That is, for each phoneme, we calculated the average rating values of words that contain this phoneme (Fig 1A). This way, the *Affective Semantic Content* (*W*_*i*_) and the error term ϵ nearly cancel out, and the average ratings are approximately associated with the potential contribution of each single phoneme (i.e. the PAV) to the rating value.

### Corpus preparation

In order to have an adequate number of repetitions and hence improve the SNR, we chose only those phonemes with a frequency of appearance higher than 30 in the database (mean frequency = 322). This led to the exclusion of 120 words that contained phonemes with a lower frequency, including those that are not a part of German phonology. Overall 12 phonemes were excluded, seven with a frequency of seven to 30 (ʒ, ɛː, |:, j, pf, tʃ, /) and five which did not belong to German phonology: $\tilde{/}$ː, dʒ, $\tilde{æ}$ː, $\tilde{ɔ}$ː, $\tilde{ɑ}$ː.

### Calculation of PAPs

For a given word W_k_ composed of n phonemes {Ph_1_, Ph_2_,…, Ph_n_} with a rating R_k_ (valence or arousal), we first defined a membership function 𝛼 as follows:

$$\left\{\begin{array}{l} Ph_{i}\in W_{k}\Rightarrow \alpha (Ph_{i},W_{k})=1\\ Ph_{i}\notin W_{k}\Rightarrow \alpha (Ph_{i},W_{k})=0 \end{array}\right\}$$

For each of the 36 phonemes in the database (Ph_i_) the *PAV* was calculated (separately for arousal and valence) as follows:

$$PAV(Ph_{i})=\frac{\sum_{j=1}^{N}\alpha (Ph_{i},W_{j})\times R_{j}}{\sum_{j=1}^{N}\alpha (Ph_{i},W_{j})}$$

Where W_j_ is the j^th^ word in the database, and N is the number of whole words (= 2574). Results are shown in S1 Table.

For each word in the database (W_k_), we then calculated its PAP (again, separately for arousal and valence) by averaging across all PAVs (see Fig 1A). The *Phonological Affective Potential* (for valence or arousal) for a given word W_k_ will then be:

$$PAP(W_{k})=\frac{\sum_{i=1}^{n}\alpha (Ph_{i},W_{k})\times \frac{\sum_{j=1}^{N}\alpha (Ph_{i},W_{j})\times R_{j}}{\sum_{j=1}^{N}\alpha (Ph_{i},W_{j})}}{\sum_{i=1}^{n}\alpha (Ph_{i},W_{k})}$$

Note that all of the following studies were conducted for the remaining number of 2574 words for which the PAP was calculated.

## Study 1: Relating words’ acoustic features and PAP

If the PAP of written words is somehow linked to emotional cues present in their phonological forms, we would expect it to be related to the acoustic features of that form.

### Method

To test the above relationship, we synthesized the words and extracted their acoustic features, focusing on a total of 11 features that are known to modulate emotional vocalization: fundamental frequency (f0; mean), sound intensity (mean and standard deviation), spectral center of gravity (mean), standard deviation of the spectrum, and sound formants (F1, F2, F3; means and bandwidths) [45,58–60].

It is worth pointing out that we deliberately opted for synthesizing the words rather than using a human speaker in order to prevent any undesired emotional prosody that might result from words’ affective meaning: Human speakers tend to pronounce words with a prosodic intonation—independently from phonological content—consistent with words’ meaning. By synthesizing the words, we distinctly separated our dependent and independent variables: PAPs (PAP_aro_ and PAP_val_) on the one side, and acoustic features on the other side. Although the artificial nature of a synthesized voice could diminish the goodness of acoustic models, a positive result would all the more support the effects in question.

### Synthesizing and acoustic analyses

We synthesized the words using the eSpeak [61] as front-end to the male voice de4 from MBROLA [62] which consists of a speech synthesizer; based on the concatenation of diphones, and of diphone databases. We abstained from the use of larger synthesis units (such as whole words or phrases as used in Variable Unit Concatenation systems) to avoid the potential effect of words’ affective content on speakers’ prosody as discussed above [63]. Words were synthesized in a fixed carrier sentence, *Das Wort … wird oft verwendet* (“The word … is often used”). The rate of speech was set at 120wpm (words per minute). All spoken words were checked for intelligibility by two male native speakers (not otherwise involved in the study). Importantly, the speakers were not provided with the word list so that they had no expectations about the words’ identity [64]. Both speakers agreed on the intelligibility of all words: speaker1 marked four words and speaker2 marked seven words as poorly synthesized; however, they found all words still intelligible. We extracted the acoustic features of words using the speech analysis software PRAAT [65]. We extracted the mean of fundamental frequency f0 (time step = .01, min = 75Hz, max = 300Hz), the mean and standard deviation of intensity (time step = .01), and the mean and the bandwidth of the first three formants F1, F2 and F3 (time step = .01) from the spectral representation of the sound. Finally, the spectral centroid (spectral center of gravity) and the standard deviation of the spectrum were computed on the basis of fast Fourier transformations (time step = .01, min pitch = 75Hz, max pitch = 300Hz).

### Results and discussion

We computed two multiple linear regression models to predict the PAP_aro_ and the PAP_val_ using the above distinctive acoustic features as regressors (N = 11). The acoustic analyses reported next were carried out on all of the 2574 words in the database. The results are summarized in Fig 1B and S2 Table. For both arousal and valence, PAPs were significantly predicted by the distinctive acoustic variables (both Ps < 0.0001), the variance accounted for being 27.9% for the PAP_aro_ and 23.7% for the PAP_val_ (both R^2^ adjusted).

Words in the database that are derived from the same stem or root morpheme (e.g. ‘*terror’* and *‘terrorize’*) are likely to have both similar phonological structure and similar semantic content. This could potentially bias the relationship between PAP and the way words sound. Hence, to ensure unbiased estimation, we selected all monosyllabic words from the database (N = 289) and repeated the above analysis steps (including new calculations of PAPs) for this subset comprising only monomorphemic words for which stem repetition was precluded. This time, the 11 acoustic variables accounted for 29.3% of the variance in PAP_aro_ and 26.6% in PAP_val_ (both R^2^ adjusted, Ps < 0.0001). The successful outcome and even larger portion of explained variance corroborates our previous results and validates the method used to uncover the effects of phonological units. We expected to obtain a better approximation for PAV (and consequently PAP) when the number of phonemes in a word is reduced, as is the case for monosyllabic words.

By showing that a considerable part of the variance in PAPs depends on the acoustic features of the spoken word forms, we could reject H0 stating that PAPs are a mere product of chance. Instead, H1 was supported: acoustic features of phonemes significantly co-determined words’ affective ratings even when they are visually presented (and silently read). This suggests that the contribution of phonological units to the ratings of words’ affective meaning—as reflected in the PAPs—emerges from a representation of acoustic properties of words in spoken form. We take this as a first support for the validity or psychological reality of the effect in question.

Having shown that PAPs of written words are significantly associated with the acoustic profile of their spoken form, we next asked whether this association is based on the words’ affective sound as assessed in two independent ways (study 2a and 2b).

The following studies were approved by the ethics committee of the Freie Universität Berlin and were conducted in compliance with the Code of Ethics of the World Medical Association (Declaration of Helsinki). All participants gave their consent (in written form for study 2a, and online for study 2b) prior to participating in the study.

## Study 2a: Measuring words’ affective sound via rating

### Stimuli

The stimuli were the 2574 words from the BAWL used in the previous analyses.

### Participants

A total of 272 participants were recruited by flyers, email contacts, and Facebook posts, who then rated the words either for valence or for arousal. Of these, 135 participants (82 females, age = 21.3 ± 4.6) rated exclusively for arousal and 137 participants (92 females, age = 23.6 ± 2.9) for valence. Participants were mostly students from the Freie Universität Berlin who received either psychology course credit or 5 Euros for their participation. All participants reported normal or corrected-to-normal vision and were native German speakers.

### Procedure

Words were presented visually. A very similar set of instructions to those used to rate the words’ affective meanings [57] was applied here, with one minor modification. Participants were instructed that they would have to suppress the meaning of words and only pay attention to their sound, and this was repeatedly emphasized through the instruction process. We also incorporated the self-assessment manikins (SAM) that were used in the ANEW study [66]. Words were randomly divided into 8 different lists each of which included about 335 items. Words were then rated on both affective sound of valence and affective sound of arousal by different groups of participants in order to exclude the possibility of mutual influence between valence and arousal ratings. The affective sound of valence was rated on a 5-point scale ranging from -2 (*sehr negativ* / “very negative”) through 0 (*neutral* / “neutral”) to +2 (*sehr positiv* / “very positive”). The 5-point affective sound of arousal scale ranged from 1 (*sehr beruhigend* / “very calming”) to 5 (*sehr aufregend* / “very exciting”). The items were randomly presented to minimize primacy or recency effects. On average, the tasks were completed in approximately 25 minutes.

### Analysis

Each word was rated by an average of 19.7 participants for valence and 20.4 participants for arousal. In order to assess the degree of agreement among raters, the Interclass Correlation (ICC) was computed for both arousal and valence ratings. Results showed a higher value for arousal (ICC = 0.43) than for valence (ICC = 0.31), indicating a rather poor degree of agreement.

Even though participants were asked to only focus on the affective *sound* of words, their ratings were likely “contaminated” by words’ semantic content, since semantic representations are automatically activated during word recognition. This was evident in the correlations between our ratings of affective sound and the original *Affective Meaning Ratings*: r = 0.32 for arousal and r = 0.22 for valence. To eliminate the undesired effect of words’ *Affective Semantic Content* from our ratings of affective sound, we opted for the most conservative approach. We first regressed the PAPs on the *Affective Meaning Ratings*—separately for arousal and valence—and used the residuals as a statistical estimate for words’ *Affective Semantic Content* (cf. Eq 1). In a next step, we regressed the estimate for words’ *Affective Semantic Content* on our ratings of affective sound and used the z-transformed residuals of this regression as independent measures of words’ affective sound. This way, the potential effect of *Affective Semantic Content* was partialed out of rating values of affective sound. The substantially weaker correlations between these “decontaminated” residuals and the original *Affective Meaning Ratings* validated our method: r = 0.1 for arousal, and r = 0.04 for valence. These two decontaminated residuals (for arousal and valence) were then used as estimates of the words’ affective sound. In the following, we refer to these two measures as *Affective Sound Ratings*; in short: AS-R_aro_ (for arousal) and AS-R_val_ (for valence).

### Results and discussion

The correlations between AS-R_aro_ and AS-R_val_ on the one hand, and PAP_aro_ and PAP_val_, on the other hand, were highly significant: r = 0.5, for arousal, and r = 0.25, for valence (both Ps < 0.0001). A similar analysis was performed for the subset of monosyllabic words (N = 289): AS-R_aro_ and AS-R_val_ were also significantly correlated with the corresponding PAP_aro_: r = 0.46, and PAP_val_: r = 0.32, respectively (both Ps < 0.0001).

These results indicate that the contribution of phonological units to words’ affective meaning ratings (i.e. PAPs) is associated with the affective sound of these words, thus providing further support for the psychological reality and the affective nature of PAPs.

## Study 2b: Predicting words’ affective sound via acoustic models

In the previous study, the poor ICC values suggested that subjective judgments about the affective sound of a word while trying to suppress its meaning can be a difficult task. In this study, we therefore aimed to provide a new measure of affective sound by using meaningless pseudowords that would allow participants to better focus on the sound. We therefore generated and presented pseudowords in auditory form and collected ratings of their affective sound. By extracting the acoustic features of pseudowords and using them as predictors we developed acoustic models capable of predicting the variation in the ratings. Such independent models can then be applied to any word-like item in auditory form to predict its affective sound solely based on its acoustic features, including the real words from the previous studies. Note that since the pseudowords had to be presented to and rated by human subjects, for this task—unlike in Study1—we used a human voice rather than a synthesizer to generate naturally sounding stimuli and to prevent potential distortion effects of sound peculiarity. However, as pseudowords lack semantic content there was no concern about the influence of meaning on emotional prosody as in the study1.

### Stimuli

To generate pseudowords representative for the phonotactics of German, we used the Wuggy algorithm [67] which generates pseudowords that match a given word template in sub-syllabic structure and transition frequencies, thus obeying a language’s phonotactic constraints. Since the pseudowords had to be spoken and rated, to avoid obscureness we restricted the list of word templates to those having up to three syllables and 10 letters. We then chose the first 1500 most frequent nouns from CELEX [68]. For each word, we adapted the program to generate five pseudoword alternatives using Wuggy’s default setting. Candidate pseudowords which differed in fewer than two letters (whether added, deleted or substituted) from the nearest real word were excluded due to their similarity to real words (Coltheart distance = 1). For words with more than one remaining pseudoword alternative, the one with a highest Levenshtein distance [69] was selected. The list of pseudowords was checked for pseudohomophones and a too high similarity to real words. Thus, 187 items were excluded: for example, *beim* (similar to the short form of “*bei dem*” = for something), *absads* (similar to the word “*Absatz*” = paragraph). In addition, because of phonotactic problems mostly caused by illegal or very rare grapheme combinations 190 items were excluded: for example Weckbeveuz, Ymiön, by two native speakers. The remaining 1123 pseudowords were selected for recording.

A professional male actor was recruited in Berlin, Germany, who was a native speaker of German. He had graduated from professional acting school and was regularly employed in radio, television, and stage work. He was paid to participate. Pseudowords were spoken in a list-like manner to prevent affective prosody and were recorded in the “Leibniz-Zentrum Allgemeine Sprachwissenschaft” in Berlin in a professional sound recording booth using a “Sennheiser MKH20” microphone and “Ultra Gain MIC-2000” preamplifier. The audio signal was recorded using the DAT-recorder “TASCAM DA20MKII” with a sampling frequency of 48 kHz and 16 bits per sample.

### Participants

A total of 169 participants were recruited by flyers, email contacts, and Facebook posts, who rated the pseudowords either for valence or for arousal. Of these, 85 participants (52 females, age = 26.7 ± 4.3) rated exclusively for arousal and 84 participants (42 females, age = 27.1 ± 3.8) for valence. Participants had the chance to win one of 10 Amazon coupons which were assigned randomly at the end of the study. All participants were native German speakers.

### Procedure

In order to afford a convenient method of sampling that was more representative of the general population, the study was conducted online using the SoSci panel [70]. Adapting the instructions used for the original BAWL ratings for written words, participants were invited to carefully listen to the presented item and evaluate how positive or negative (in the case of valence) and how exciting or calming (in the case of arousal) the pseudowords sounded. During the rating process, a “replay” button was offered to provide participants with the opportunity of repeated listening to each presented item. We also incorporated the self-assessment manikins (SAM) that were used in the ANEW study [66]. Importantly, participants were instructed to give their ratings solely based on the sound aspect of items and not based on their similarity to real words. In order to prevent participants from giving a rating for a similar sounding word, a button labeled “concrete word” was provided next to the rating scale, and participants were instructed to use it in case an item might remind them of a German word. 28 items labelled as “concrete word” by more than 50% of participants were then excluded, leaving 1095 items for further analysis. Pseudowords were randomly divided into 4 different lists, each including about 280 pseudowords. The order of presentation was pseudorandomized for each participant. On average, the task was completed in approximately 15 minutes.

### Analysis

**Fig 2:**
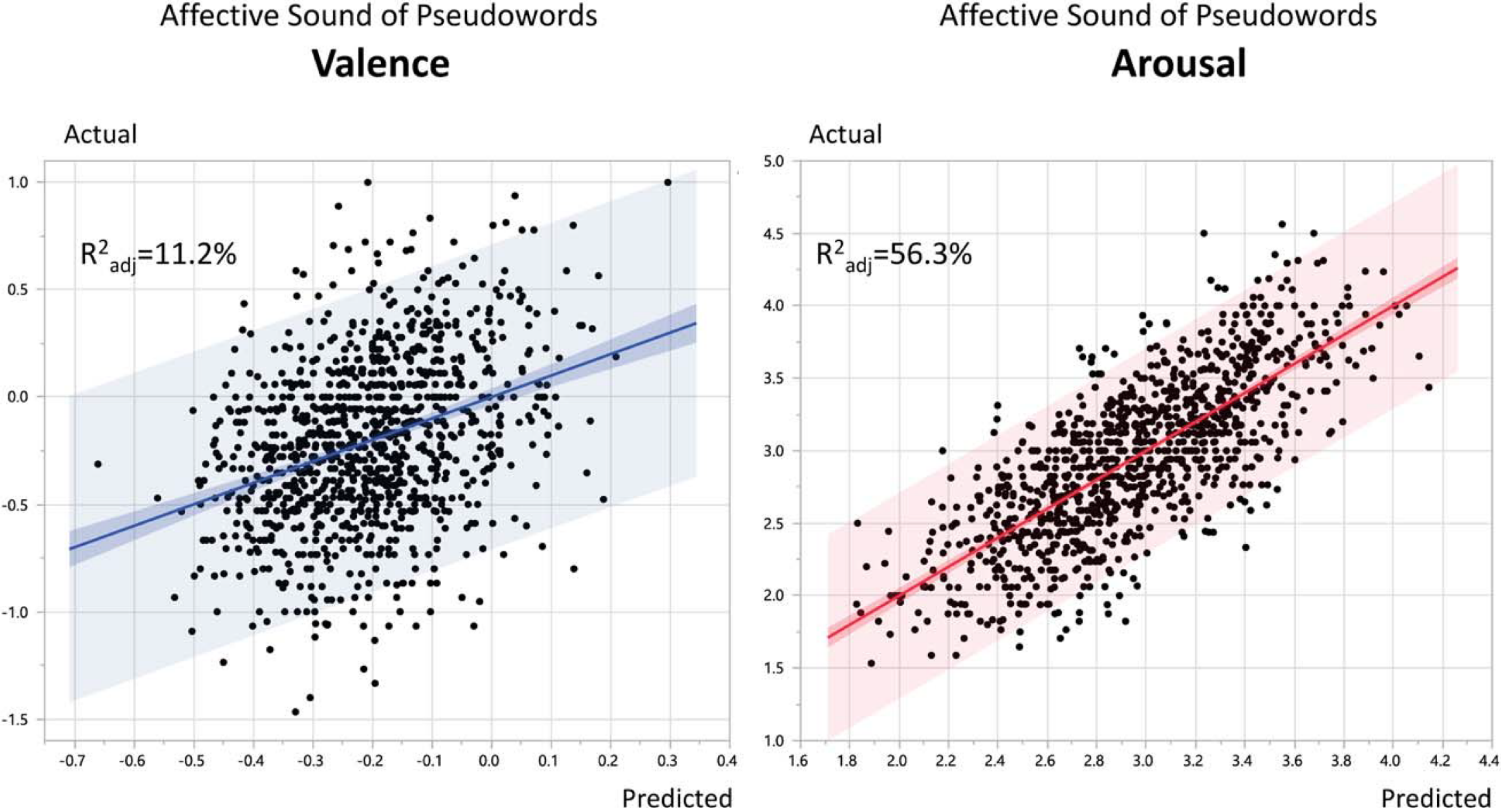
Acoustic features of pseudowords (N = 11) significantly predicted the ratings of their affective sound: 11.2% for valence (left) and 56.3% for arousal (right).

Affective ratings were obtained for 1095 pseudowords with 17 ratings per item on average (17.2 for arousal, and 17.5 for valence). We extracted the 11 acoustic features from the spoken pseudowords (see study 1) and performed two multiple regression models using them as predictors of the ratings separately for arousal and valence. These features accounted for 56.3% of the variance in arousal ratings and 11.2% for valence (both R^2^ adjusted, Ps < 0.0001, Fig 2).

Since our ultimate goal was to predict the affective sound of real words, in order to assess how the results of the above models generalize to an independent data set (i.e. real words), we used two-fold cross-validation. The dataset was randomly shuffled into two subsets with equal size one for training and one as a test set, and vice versa. Model accuracy for each run was 57.3% and 52.6% (both R^2^ adjusted, Ps < 0.0001) for the arousal model, and 10.1% and 9.9% for the valence model (both R^2^ adjusted, Ps < 0.0001). These are very robust results in terms of explained variance compared to the original models.

### Results and discussion

The degree of agreement among raters, compared to the results of study 2a, was considerably higher for both valence (ICC = 0.61) and arousal (ICC = 0.86). The substantial amount of variance accounted for in our regression model for arousal indicates that the affective sound of word-like stimuli could be mapped out in terms of their acoustic cues; a strong evidence for acoustic features to possess affective value on their own. A closer look at the variation in ratings revealed a smaller relative standard deviation for valence (13%) than for arousal (18%), suggesting a lower consensus among participants when rating valence. The considerably higher degree of explained variance for arousal as compared to valence supports the idea that speech sounds primarily signals the sender’s arousal state (and their valence state only to a smaller degree) [59,71]; we will discuss this finding more fully later in this article.

We next took the two acoustic models (i.e. the linear equations in S3 and S4 Tables) resulting from the pseudoword data and applied them to the extracted acoustic features of the words in the database to predict words’ affective sound. We refer to these predicted values for words’ affective sound as *Affective Sound Predicted*; in short: AS-P_aro_ (for arousal) and AS-P_val_ (for valence).

The obtained predicted values for words’ affective sound (i.e. AS-Ps) were then compared with PAP: The AS-P_aro_ and the AS-P_val_ of words were significantly correlated with the PAP_aro_: r = 0.47, and with the PAP_val_: r = 0.36, respectively (both Ps < 0.0001). Again, similar results were obtained for monosyllabic words (N = 289): AS-P_aro_ was significantly correlated with PAP_aro_: r = 0.45, P < 0.0001, and AS-P_val_ was significantly correlated with PAP_val_: r = 0.42, P < 0.0001.

These significant associations between words’ PAP and their affective sound of words—independently predicted from acoustic features—add additional support for our H1. In addition to the direct correlation between PAPs and words’ affective sound, as captured by AS-Ps (AS-P_aro_ and AS-P_val_), we tested the relationship between AS-Ps and those proportions of variance in PAPs that we could account for by means of acoustic features in the first analysis: that is, the predicted values for the PAP_aro_ and the PAP_val_ in the first multiple regression models (Study1, Fig 1B) that were calculated with the same acoustic variables as regressors. Results showed high correlations between AS-P_aro_ and the predicted values for PAP_aro_: r = 0.88, and between AS-P_val_ and the predicted values for PAP_val_: r = 0.71 (both Ps < 0.0001). This suggests that the PAPs are based on the same distinctive acoustic features that participants used to evaluate the affective sound of pseudowords, thus, again, providing strong evidence for the association between PAPs and affective sound, and that a significant portion of variance in the ratings of words’ affective meaning is due to how words affectively sound.

Furthermore, we tested the reliability of our two different measures of words’ affective sound as described in Study 2a and Study 2b, to investigate their consistency in capturing the same concept. For this, we compared the values resulting from these completely independent methods for measuring words’ affective sound. Results showed significant correlations between the measure of affective sound based on the direct rating value (i.e. AS-R, Study 2a) and the predicted values of affective sound based on acoustic features (i.e. AS-P, Study 2b) for both arousal: r = 0.56, P < 0.0001, and valence: r = 0.49, P < .0001. These results, together with the fact that PAPs are associated with words’ affective sound, provide firm support for our H1 stating that the affective meaning of words is shaped by both words’ semantic content and (implicit) affective sound.

## Analysis of words’ acoustic profiles

Having shown a robust association between PAPs and two independent measures of affective sound (AS-R and AS-P), we continued with a more fine-grained analysis and asked whether the underlying acoustic features shaping PAP and both AS-R and AS-P, do so in identical or differential ways for these different measures. Thus, we examined the direct relationships between each acoustic feature and the PAP on the one hand, and our two measures of affective sound (i.e. the AS-R from study 2a and the AS-P from study 2b) on the other.

### Method

**Fig 3:**
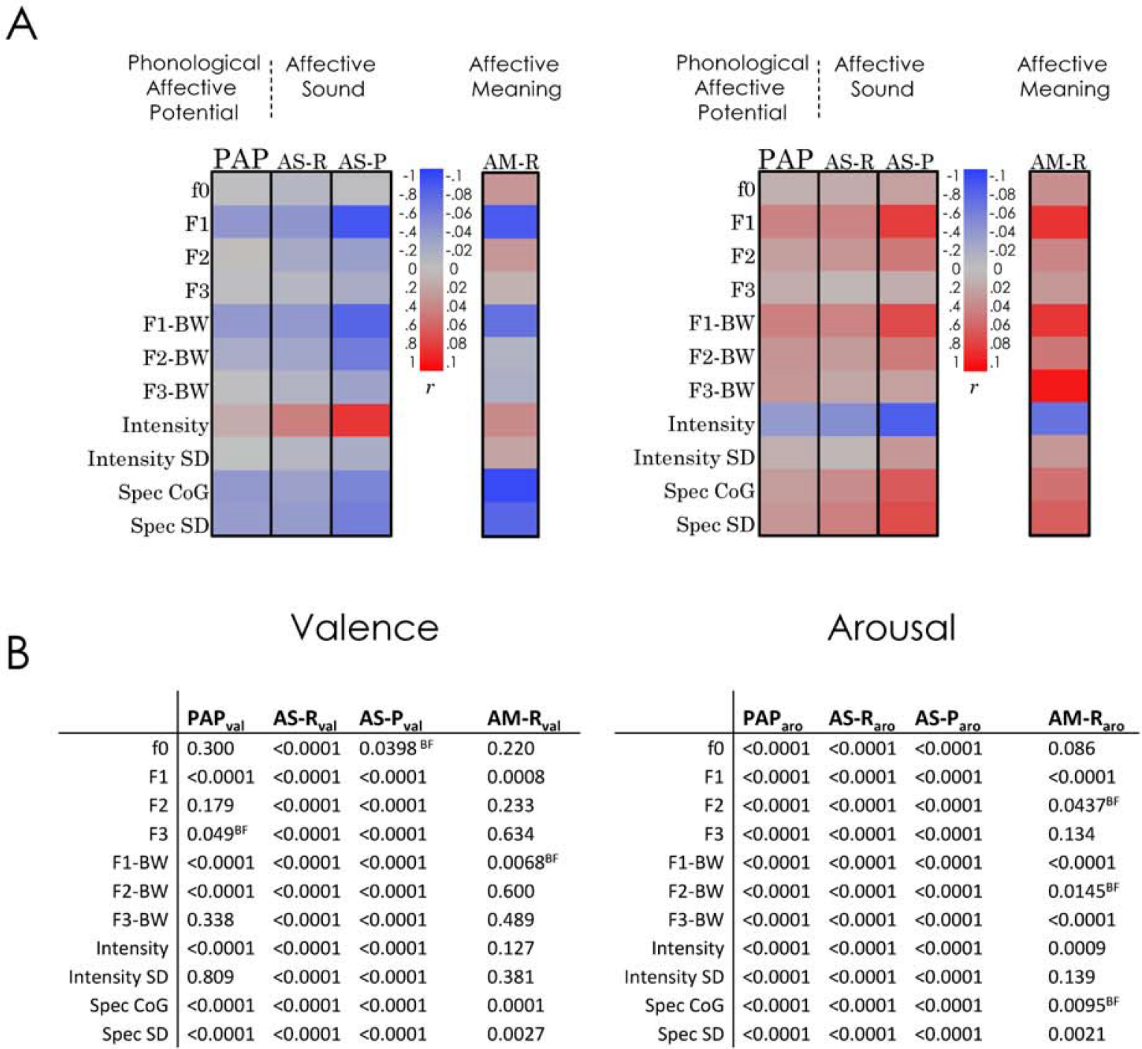
**A)** Acoustic profiles were constructed (using correlation cell plot) based on the strength and direction of correlations between the estimated effect of words’ phonology on the evaluation of their affective meaning (i.e. Phonological Affective Potential: PAP), the two measures of words’ affective sound (i.e. Affective Sound-Ratings: AS-R [study 2a], Affective Sound-Predicted: AS-P [study 2b]), and ratings of words’ affective meaning (i.e. Affective Meaning-Ratings: AM-R) on the one hand, and 11 acoustic variables on the other hand (left for valence, right for arousal). Acoustic features that significantly correlated with the PAP, AS-R, AS-P, and AM-R always show associations in the same direction, suggesting that acoustic features underlying the affective sound of words contribute in similar ways to the constitution of affective meaning of these words. **B)** The correlation probabilities are shown in the table. Correlations not surviving Bonferroni correction for multiple comparisons are marked with “BF” (Bonferroni Failed). Abbreviations: BW = Bandwidth, SD = standard deviation, Spec = Spectral, CoG = Centre of Gravity, r = correlation coefficient.

We constructed acoustic profiles based on the strength and direction of correlations between each of 11 acoustic variables with PAP, AS-R, and AS-P (see Fig 3, see S5 Table for correlation coefficients).

### Results and discussion

For the arousal dimension, all single correlations (N = 3x11) were highly significant (Ps < 0.0001). Notably, the correlations between each acoustic variable and PAP_aro_ were always in the same direction as correlations between this specific acoustic variable and both measures of affective sound for arousal (i.e. the AS-R_aro_, and the AS-P_aro_) resulting in highly similar acoustic profiles for all three measures (Binomial test: X ~ B (11, 0.5), p (X≥11) = 0.0005). A similar pattern was observed for valence. The PAP_val_ was significantly correlated with seven acoustic variables, and, importantly, these correlations were, again, always in the same direction as for the acoustic variables and both measures of affective sound for valence (i.e. the AS-R_val_, and the AS-P_val_) again resulting in highly similar acoustic profiles for all of three measures (Binomial test: X ~ B (7, 0.5), p (X≥7) = 0.007).

All correlations in the acoustic profile of arousal remained significant after Bonferroni correction for multiple comparisons. For the acoustic profile of valence, however, the correlation between the third formant (F3) and PAP_val_ did no longer reach statistical significance after Bonferroni correction. But, still, acoustic profiles for all of three measures (AS-R_val_, AS-P_val_, PAP_val_) remained highly similar (Binomial test: X ~ B (6, 0.5), p (X≥6) = 0.015).

These results go beyond the simple relationships between the PAPs and the affective sound of words (as captured by AS-P and AS-R); moreover, they show that the acoustic features that underlie PAPs contribute in very similar ways to the perception of words’ affective sound. We interpret this as strong support for PAPs being determined by affective perceptual cues within phonological word forms.

## The direct effect of sound on words’ affective meaning

Here, we asked whether the contribution of words’ (implicit) sound to words’ affective meaning can be directly observed at the level of original valence and arousal ratings in the database: that is, before estimating the effect through our statistical operationalization for the PAP. In other words, if an *Affective Meaning Rating* consists of *Affective Semantic Content* and *Phonological Affective Potential* (PAP), as formulated in Eq 1, we would expect that the same acoustic features shaping the PAP should be reflected, though to a lesser degree, in *Affective Meaning Ratings*. That is the effect of words’ acoustic features on words’ affective meaning should be observable directly at the level of *Affective Meaning Ratings*.

### Method

We constructed acoustic profiles for *Affective Meaning Ratings* by calculating correlations between *Affective Meaning Arousal-Ratings* (in short AM-R_aro_) and *Affective Meaning Valence-Ratings* (in short AM-R_val_) and each of the acoustic variables across all words in the database (N = 2574).

### Results and discussion

From the total of 11 acoustic variables, eight variables in the acoustic profile for arousal (five variables after Bonferroni correction) and four in the acoustic profile for valence (three variables after Bonferroni correction) were significantly correlated with AM-R_aro_ and AM-R_val_, respectively (Fig 3 and S5 Table). Most importantly, those acoustic features that significantly correlated with AM-R_aro_ and AM-R_val_ always showed an association in the same direction as the one between the acoustic features and respective PAP, as well as both measures of words’ affective sound (i.e. the AS-R and AS-P). Again, these results support the direct relationship between words’ acoustic features and ratings of affective meaning.

Together with our previous findings, these data suggest that the process of meaning making is not solely determined by arbitrary mappings between words’ phonology and concepts they refer to. Rather, words’ acoustic profiles provide affective perceptual cues that language users implicitly use to construct words’ affective meaning.

## Acoustic phonetic cues underlying the effect of sound on meaning

Revealing the perceptual acoustic cues likely underlying the effect of implicit sound on affective meaning, we performed further analyses to explore phonetic features potentially causing this effect.

The consistently negative correlations between sound intensity and each of the four arousal-based measures: PAP_aro_, AS-R_aro_, AS-P_aro_, and AM-R_aro_ (Fig 3) deserves a more detailed discussion as arousal usually increases with sound intensity when the latter is experimentally manipulated. Note that all words and pseudowords were spoken with the same loudness, thus differences in sound intensity have to be tracked back to specific phonetic features of the words in the database.

### Long vs. short vowels

**Fig 4:**
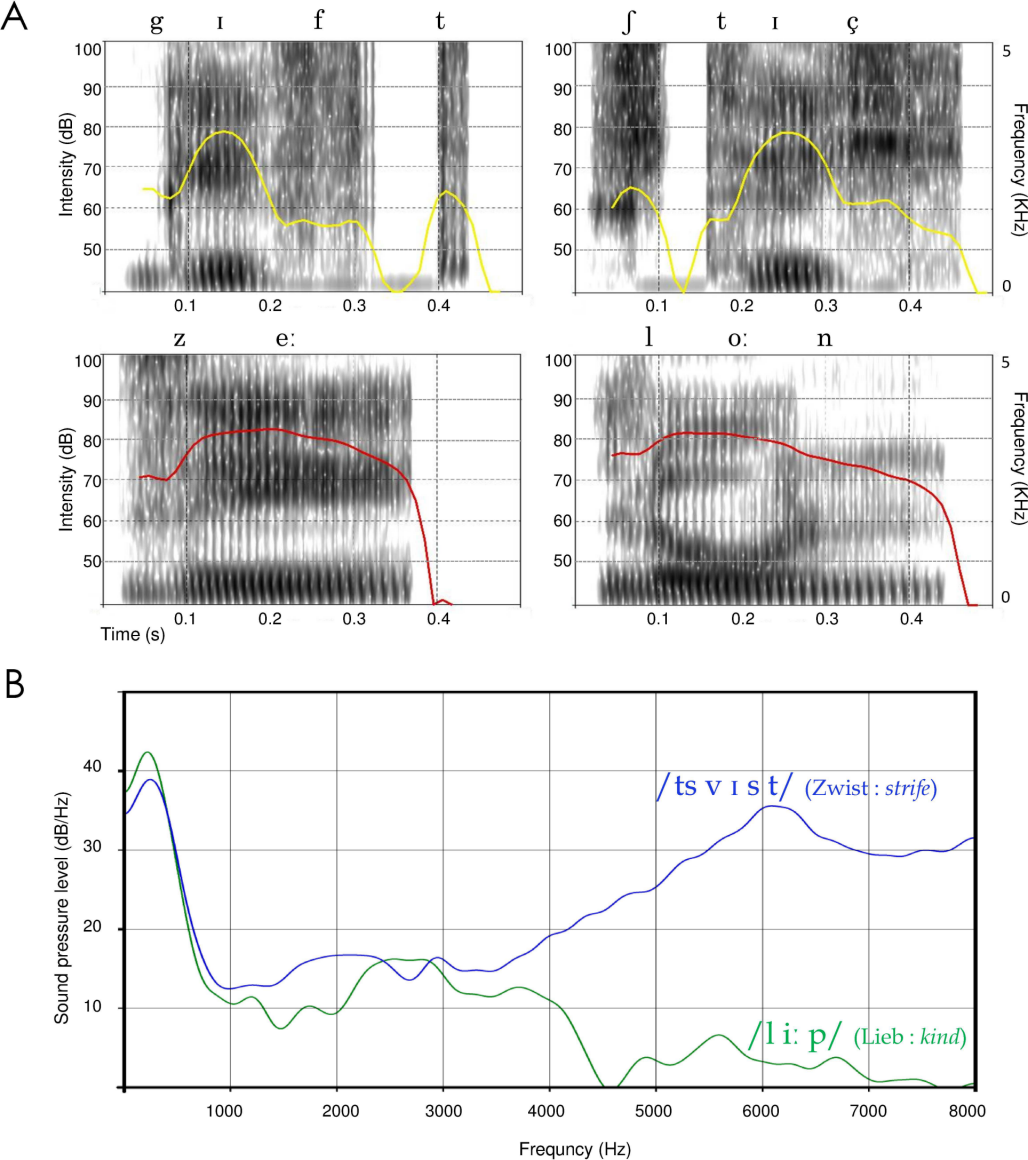
**A)** The time course of sound intensity for the words “Gift /g ɪ f t/ *(gift)*” and “Stich /ʃ t ɪ ç/ *(stab)*” (top, yellow lines) compared to their counterparts “See /z e:/ (*lake*)” and “Lohn /l oː n/ (*wage*)” (bottom, red lines). Short vowels, plosives, and voiceless consonants (as in “Gift” and “Stich”) possess smaller integrals of sound energy, whereas sustained high amplitude (see red lines) results in larger sound intensity. This relationship between phonetic features and sound intensity, together with the relationship between sound intensity and ‘affective sound’ of words, explains the harsh sound of words containing short vowels, plosives, and voiceless consonants. **B)** Spectral analysis shows that hissing sibilants in a word increase the sound’s center of gravity (i.e. the magnitude-weighted mean of the frequencies present in the signal), which makes words including this category of phonemes sound harsh and negative (blue line Zwist /ts v ɪ s t/ *(strife)* vs. green line Lieb /l iː p/ *(kind)*).

A closer look at the spectrograms reveals that words with the highest sound intensity tend to include long vowels (e.g. Lohn /l oː n/ “*wage*”, See /z e:/ “*lake*”, see Fig 4A). To systematically examine this potential relationship, we defined a Vowel Length Index as the average vowel length (short = 1, long = 2) over the word’s syllables. This Vowel Length Index was significantly correlated with sound intensity across all words in the database: r = 0.28, P < 0.0001, suggesting a systematic relationship between the two measures. With regard to affective perception, note that long vowels are produced through a release of air from the mouth for an extended period of time which is a behavior similar to slow (vs. rapid) breathing that, in turn, is associated with decreasing (vs. increasing) arousal [72,73]. This relationship between affective states and sound duration is also stressed in the motivation-structural rules hypothesis [74] stating that calls produced by mammals in aggressive circumstances, termed *barks* or *grunts*, are generally of shorter duration than those produced in appeasement contexts. On the other hand, at the spectrogram level, the sustained high amplitude for long vowels causes a larger integral of energy for the whole sound envelope leading to the negative correlation between arousal and sound intensity (see Fig 4A). Note also that the variation of intensity of sound over time (Intensity-SD) accordingly displays a positive correlation with arousal.

A comparison between the PAV of short vowels and their long counterparts (see S1 Table) revealed the same pattern: each of the short vowels was perceived as “more arousing” than its long counterpart: PAV_aro_(/a/) > PAV_aro_ (/aː/), PAV_aro_ (/ɔ/) > PAV_aro_ (/oː/), PAV_aro_ (/ʊ/) > PAV_aro_ (/uː/), PAV_aro_ (/ɪ/) > PAV_aro_ (/iː/), PAV_aro_ (/ɛ/) > PAV_aro_ (/ɛː/). A very similar pattern was revealed for valence values calculated for short and long vowels; with short vowels being more “negative” than their long counterparts–except for the short and long vowels /ɔ/ and /oː/, for which all calculated values were very close to zero.

In addition, short vowels tend to be followed by more consonants (i.e. more complex consonant clusters) than long vowels, and this complexity of subsequent consonant clusters may also hold partly responsible for the observed correlation between vowel length and arousal.

### Voicing

Another phonetic feature directly related to sound intensity is ‘voicing’. Voiced consonants are accompanied by vocal cord vibration that leads to an increase in sound energy compared to their voiceless counterparts. In order to explore the relationship between voicing and the affective sound of words, we defined a phonetic cue based on the relative proportion of voiced consonants to all consonants in a word. This phonetic cue of voicing was significantly correlated with sound intensity, r = 0.38 (P < 0.0001), and also with both measures of affective sound: AS-R_aro_: r = -0.51, and AS-R_val_: r = 0.49, as well as AS-P_aro_: r = -0.57, and AS-P_val_: r = 0.62, (all Ps < 0.0001).

These results indicate that voiceless consonants sound on average more arousing and negative than voiced consonants, which, in turn, appear to make words sound softer and more pleasing.

### Plosive consonants

Among words with the lowest sound intensity, many include plosive consonants (e.g. Gift /g ɪ f t/ “*gift*”, Stich /ʃ t ɪ ç/ *“stab”*). The interruption and explosive release of the air stream in the pronunciation of plosive sounds can be associated with a higher level of arousal, but at the same time, during a stop closure, there is very little acoustic energy. This may explain the lower level of sound intensity (and a higher level of arousal at the same time) for words that include this type of phonemes (see Fig 4A).

Similar to voicing, we defined a phonetic cue indicating the relative proportion of plosive consonants to all consonants in a word. This phonetic cue was significantly correlated with sound intensity: r = -0.26 (P < 0.0001) and with both affective sound measures, AS-R_aro_: r = 0.2, and AS-R_val_: r = -0.16, as well as AS-P_aro_: r = 0.17, and AS-P_val_: r = -0.19, (all Ps < 0.0001), reflecting that while plosives reduce sound energy, they also play a significant role in making the sound (moderately) more negative and arousing.

### Hissing sibilants

In addition to sound intensity and in line with previous findings on vocal expression of emotion [58–60], first formant (F1) and spectral centroid (CoG) appeared to be the dominant features explaining the largest part of variance in words’ affective sound, showing a significant effect even at the level of direct ratings for words’ affective meaning. A larger high-frequency energy and raising of the first formant are typical characteristics of hissing sibilants (alveolar fricatives and affricates, e.g. /s/, /z/, /ʃ/) which are strongly stressed consonants produced by a high-velocity jet of air against the teeth (see Fig 4B). This results in a literally high-arousing hissing sound, which may account for the cross- and paralinguistic use of these sounds for attracting the attention of others (e.g. “psst!”) as well as for their prominent deployment in literature as a stylistic device for cacophony.

Similarly, it is the presence of such a hissing sound following a short vowel that makes the small, but striking difference at the phoneme level between two words referring to one and the same concept from a very basic domain of physical human experience, out of which one is considered rather vulgar and rude, while the other seems more childish and polite: ‘*piss’* vs. ‘*pee’*.

## General discussion

The present data demonstrate that words’ affective meaning, as reflected in evaluative ratings, is co-determined by words’ acoustic-phonetic features. Overall, the results of our computational approach and acoustic analyses, together with the data from the behavioral studies, provide strong support for the hypothesis that phonemes possess affective potential based on their spectro-temporal acoustic features known to modulate emotional vocalization. These results emphasize the iconic nature of the relationship between the (implicit) sound of a phoneme and its affective quality on the one hand and affective meaning of words comprising these phonemes on the other.

As outlined in the introduction, with this study we addressed three major issues generally involved in previous research on iconicity. First, by focusing on a representative number of real words—instead of pseudowords—, our novel results improve the understanding of the effect of implicit sound on the process of meaning making for natural words, in particular concerning their affective meaning. We showed that not only specific sound profiles of words have an affective quality, but also that this quality implicitly influences language users in their final emotional judgment about the meaning of words. Secondly, our behavioral studies and acoustic analyses helped to overcome a major limitation of previous work showing that the relation between affective sound and meaning reflects more than just some statistical regularities within the language (i.e. systematicity) to which language users might be sensitive. Rather, our data suggest that the sound shape of words possesses an inherent affective quality (i.e. iconicity) based on acoustic features that are known to modulate nonverbal emotional communication. Finally, investigating the role of affect and affective meaning of words, we moved beyond the narrow focus on single, limited semantic concepts (see also [19]), which enabled us to test sound-meaning correspondences across a wide range of words, representative of the entire lexicon.

Importantly, the iconic affective potential of phonemes (i.e. PAP), as suggested by our results, contributes to the process of affective meaning making even when words are visually presented and silently read. Note that visual word recognition generally involves the activation of phonological codes [48–50] and language users appear implicitly influenced by affective sound of visually presented words when evaluating the affective meaning of these words.

### Valence vs. arousal

Overall, our results were generally stronger for arousal than for valence. This finding aligns with a number of studies on the acoustic properties of emotional speech and hence provides support for an “acoustic arousal dimension”. That is acoustic speech properties provide vocal cues to the level of arousal, above that of valence [56,59,60,75]. Reviews of earlier attempts to decode emotional significance from vocal cues commonly failed to identify a set of vocal features that reliably differentiate between the levels of valence. Arousal is generally understood as related to a physiological state of being reactive to a stimulus, and it appears plausible that this could be reflected in the vocal behavior of the sender and thus extend to acoustic features of the speech signal. Valence, on the other hand, involves higher order, cognitive, and evaluative processes that are less likely to be detectable at such a basal sublexical level [56,76–78], thus corresponding less well than arousal to any consistent acoustic mapping.

### Phonetic features

Our analyses of acoustic cues revealed some specific phonetic features as potential candidates of carrying the effect of sound on meaning. Short vowels, compared to their long counterparts, can make words sound more negative and arousing. Also, voiceless consonants, hissing sibilants, and—to some extent—plosives, can significantly contribute to making a word more negative and arousing–as our data suggest, both at the level of sound and perceived meaning.

It is worth noting that these phonetic cues may not be universal across different languages, as not all languages display systematic variations of some of the phonetic features that we focused on (e.g. long/short vowels). Users might rely on different phonological/acoustic affective cues in different languages depending on their phonemic inventory and phonotactic rules—to be investigated in future research on the topic.

### Measuring the affective sound of words

Our two studies present, for the first time, two different methods for assessing words’ affective sound that can be used in future studies investigating the interaction of words’ affective sound and meaning. The poor ICC values for the first method (Study 2a) indicate the difficulty of subjective judgments of the implicit sound of a visually presented word independently of its meaning. Even though we attempted to decontaminate these rating values from the effects of semantic content, this method possesses serious limitations and the poor ICC values call for cautious interpretation of these results.

In contrast to the first method, by using pseudoword material in auditory form in the second study (study 2b), we could largely overcome the limitations of the first approach and provide a better way for assessing words’ affective sound, as indicated by the considerably larger ICC values for the pseudoword ratings. Thus our approach based on pseudowords may represent a reliable proxy for words’ affective sound in future research.

### Alternative interpretation

The present approach aimed at describing the relation between words’ phonology and affective ratings in most basic ways, but our findings might also fit well into proposals concerning iconicity and the organization of the vocabulary: Rather than reflecting a direct, forward influence of acoustic features on affective ratings, PAVs, determining PAPs for words in our data might instead, reflect the systematic occurrence of specific phonemes in words of specific affective meaning (in terms of arousal and valence levels) across the vocabulary of a language. This is because PAVs are is computed as the average of affective ratings of words comprising a given phoneme. In that case, our data establishing close relations between PAP (or PAV) and acoustic features would help explain an apparent systematic distribution of phonemes across the vocabulary as a function of semantic affective values of words: An iconic relation would link affective attributes of the percept or the basic linguistic sign at the phoneme level with affective semantic meaning at the lexical level—adding an internal to the external relation between the signifier and the signified that would have contributed to the evolution of the vocabulary according to affective iconicity.

### Limitations and future research

Our study is the first to demonstrate an association between affective sound and meaning for real words and across a language lexicon. While providing important novel evidence, it also has limitations future research may attempt to overcome.

When modeling our alternative hypothesis H1, for the sake of simplicity and in the absence of a theoretically or empirically justified theory, we opted for a simple additive method (see Eq 1). It is, however, possible that words’ *Semantic Content* and PAP have an interactive effect on ratings of affective meaning. Similarly, the role of each phoneme in a word for contributing to the PAP might be differentially weighted depending on its position in the word (see Eq 2). Applying more sophisticated methods such as machine-learning-based regressors (e.g. [79,80]) might help integrate the large number of potentially influential factors into more complete and accurate models of the process of evaluative rating.

Also, a number of the acoustic features we used are measured as average frequencies, which precludes the use of dynamic sound features (e.g. spectral flex). Employing other methods based on dynamic changes of the sound signal might increase the accuracy of acoustic models predicting ratings of words’ affective sound. A more sophisticated approach, for instance, might use the matrix of the spectrogram to quantitatively represent the sound envelope. Since the length of the audio signal (i.e. the length of words or pseudowords) differs for each item, the challenge of such an approach would be to find an appropriate method to classify the (pseudo)words’ affective sound based on a series of independent variables, the number of which depends on physical signal length.

Alternatively, our acoustic analysis can be complemented by the use of phonetic categories (e.g. voiced/voiceless, obstruent/sonorant, etc.) to relate these categories to the effect of sound on words’ affective meaning. In a simple phonetic approach, each phoneme in a word will represent a vector of phonetic features. Consequently, an entire word—comprising different phonemes—can be described as a concatenation of vectors of phonetic features, which can be used to calculate the contribution of any phonetic feature to the affective sound of words; in a similar fashion to our approach concerning PAVs. A practical approach concerning the use of phonetic features instead of acoustic variables would be the use of phonological cues defined as the proportion of consonants with particular manner and place features, and the average height and position of vowels (as provided in [81]). These cues can be used in the same way as our acoustic variables to identify phonological features underlying the PAPs. An advantage of this method would be the simple classification of the phonological construction of a word and its contribution to the sound to meaning relation. Our initial investigation has shown that such phonological cues can account for a significant portion of variance in the PAPs (25% for arousal, and 15% for valence), with the proportion of voiced consonants, and the average of vowel roundedness in a word being the most important predictors for both models of arousal and valence.

Another approach for measuring the affective sound of words can make use of the insights of sonority theory [82,83]. For this, each word can be assigned a sonority score which may also systematically contribute to affective (and aesthetic) ratings, as supported by recent findings concerning ratings of the aptness of metaphors and the beauty of words [79,84].

### Practical applications

Our findings on the effects of implicit sound on affective meaning, and specifically our acoustic model for measuring the affective sound of words effectively suggest a method for constructing words and pseudowords associated with specific affects (positive/negative, arousing/calming) or emotions (e.g., fear, disgust), which can have broad applications in various contexts from marketing and advertising to art and literature. For instance, in the field of product and brand naming, previous work has shown that the sound of a product’s name can in general set and modify consumer expectations about the likely attributes of the products [5,85] and that names with negative sounds were least preferred regardless of product category [86]. Here, our method for assessing the affective sound of words based on its acoustic features could provide a substantial improvement to previous work, which was usually based on the manipulation of a limited group of sounds (e.g. front vs. back vowels). Likewise, in artistic contexts, such as film, literature, and in particular, poetry, our method could be applied to evoke and verify particular emotional effects by use of words that possess specific implicit affective sounds.

Poetry is probably the best example of a sound meaning interaction: while it is inherently concerned with the expression and elicitation of emotions [5,56,87,88], it is deeply rooted at the perceptual level in the domain of sound [5,89–91]. Indeed, poetry has always artfully deployed sound patterns to shape order, to create a new layer of meaning, and to emphasize the affective meaning in a text. With the present study we provide a complementary method to previous attempts for analyzing poetic texts at the sublexical level [55,56,90–94], and for further examination of the influence of sound structure on affective and aesthetic reactions to verbal material intended to elicit a certain emotional impact in readers, such as advertisements, political speeches or manifests.

## Conclusion

The present studies provide novel results on the contribution of the implicit sound of a word to its affective meaning. Our findings have the potential to shed new light on various unanswered questions regarding the evolution, organization, and processing of human language by drawing attention to the role of affect as well as by substantiating the psychological reality of iconicity in everyday language. These new insights may pave the way for further cross-linguistic investigations, as well as the detailed study of the neural substrates underlying the effect of phonology and sound-meaning interaction in language use; a phenomenon creatively exploited particularly by Poe and other poets throughout history.

## Supporting information

S1 Database Database_AM_AS_PAP. (XLSX)

S1 Table (DOCX)

S2 Table (DOCX)

S3 Table (DOCX)

S4 Table (DOCX)

S5 Table (DOCX)
