# Supplementary material for: Why 'piss' is ruder than 'pee'? The role of sound in affective meaning making
Source: PLoS One. 2018 Jun 6;13(6):e0198430. doi: 10.1371/journal.pone.0198430 (PMC5991420; doi:10.1371/journal.pone.0198430)
Supplement: S1 Table — (DOCX) [file pone.0198430.s002.docx]

| **Phoneme** | **Number of**  **Phonemes** | **PAV-Arousal** | **PAV-Valence** |
| --- | --- | --- | --- |
| a | 660 | 2.771966 | -0.0592 |
| ə | 1533 | 2.694517 | 0.078447 |
| pf | 30 | 2.601649 | 0.087647 |
| ts | 243 | 2.89617 | -0.17775 |
| aː | 418 | 2.748728 | 0.009111 |
| b | 417 | 2.681068 | 0.174536 |
| au | 164 | 2.643705 | 0.014397 |
| d | 280 | 2.725176 | -0.00215 |
| eː | 316 | 2.732284 | 0.128438 |
| ɛ | 458 | 2.82648 | -0.03215 |
| f | 493 | 2.795027 | 0.068395 |
| g | 374 | 2.811771 | -0.00069 |
| h | 144 | 2.759515 | 0.171893 |
| iː | 397 | 2.815113 | 0.146776 |
| ɪ | 398 | 2.815126 | 0.11832 |
| k | 516 | 2.787631 | 0.093785 |
| l | 895 | 2.70632 | 0.033052 |
| m | 501 | 2.718058 | 0.039845 |
| n | 1308 | 2.756322 | 0.014857 |
| ŋ | 188 | 2.774024 | 0.180341 |
| oː | 368 | 2.773825 | -0.03117 |
| ɔ | 223 | 2.805409 | 0.003907 |
| p | 339 | 2.812958 | -0.01111 |
| r | 1324 | 2.829352 | -0.01325 |
| s | 345 | 2.813455 | -0.10704 |
| ʃ | 285 | 2.832619 | -0.06539 |
| t | 1112 | 2.799762 | 0.015284 |
| uː | 176 | 2.807533 | 0.269463 |
| ʊ | 306 | 2.824526 | -0.04343 |
| v | 224 | 2.771459 | 0.121828 |
| ai | 274 | 2.761602 | 0.133806 |
| x | 207 | 2.755794 | 0.022846 |
| ɔy | 55 | 3.053575 | -0.32425 |
| yː | 38 | 2.809578 | 0.155573 |
| ʏ | 39 | 2.704151 | 0.427843 |
| z | 279 | 2.618919 | 0.214624 |

**S1: The calculated values of *Phonological Affective Value (PAV)* for both valence and arousal for a list of 36 different phonemes in IPA.**
