# Supplementary material for: Why 'piss' is ruder than 'pee'? The role of sound in affective meaning making
Source: PLoS One. 2018 Jun 6;13(6):e0198430. doi: 10.1371/journal.pone.0198430 (PMC5991420; doi:10.1371/journal.pone.0198430)
Supplement: S2 Table — (DOCX) [file pone.0198430.s003.docx]

**Summary of Fit for PAP_aro_:**

RSquare: 0.282132

RSquare Adj: 0.27905

Root Mean Square Error: 0.021966

Mean of Response: 2.7685

Number of Observations: 2574

| **Term** | **Estimate** | **Std Error** | **t Ratio** | **Prob>\|t\|** |
| --- | --- | --- | --- | --- |
| Intercept | 2.73667 | 0.03980 | 68.75 | <.0001 |
| F0 | 0.00035 | 0.00009 | 3.63 | 0.0003 |
| F1-Mean | 0.00002 | 0.000003 | 7.80 | <.0001 |
| F2-Mean | 0.00001 | 2.9e-6 | 3.93 | <.0001 |
| F3-Mean | 0.00001 | 7.6e-6 | 1.67 | 0.0958 |
| F1-Bandwidth | -0.00149 | 0.00038 | -3.92 | <.0001 |
| F2-Bandwidth | -0.00012 | 0.00019 | -0.64 | 0.5250 |
| F3-Bandwidth | 0.00005 | 5.8-6 | 8.70 | <.0001 |
| Intensity | 0.00001 | 5.7e-6 | 1.92 | 0.0546 |
| Intensity - SD | 0.00004 | 6.1e-6 | 7.40 | <.0001 |
| Spectral CoG | -3.7e-6 | 4.2-6 | -0.87 | 0.3825 |
| Spectral SD | 1.6e-6 | 2.2e-6 | 0.73 | 0.4638 |

**Summary of Fit for PAP_val_:**

RSquare: 0.24034

RSquare Adj: 0.23708

Root Mean Square Error: 0.03267

Mean of Response: 0.035021

Number of Observations: 2574

| **Term** | **Estimate** | **Std Error** | **t Ratio** | **Prob>\|t\|** |
| --- | --- | --- | --- | --- |
| Intercept | 0.10262 | 0.05913 | 1.74 | 0.0828 |
| F0 | -0.00004 | 0.00014 | -0.33 | 0.7440 |
| F1-Mean | -0.00006 | 5.5e-6 | -11.75 | <.0001 |
| F2-Mean | 0.00002 | 4.3e-6 | 6.20 | <.0001 |
| F3-Mean | 7.1e-6 | 0.00001 | 0.62 | 0.5335 |
| F1-Bandwidth | -0.00103 | 0.00056 | -1.84 | 0.0666 |
| F2-Bandwidth | 0.00055 | 0.00029 | 1.86 | 0.0634 |
| F3-Bandwidth | -0.00005 | 8.6-6 | -6.71 | <.0001 |
| Intensity | 3.8e-6 | 8.6e-6 | 0.45 | 0.6537 |
| Intensity - SD | 0.00003 | 9.1e-6 | 3.76 | 0.0002 |
| Spectral CoG | -2.8e-5 | 6.3e-6 | -4.42 | <.0001 |
| Spectral SD | -1.8e-6 | 3.4e-6 | -0.55 | 0.5816 |

**S2: Summary of regression models for PAP_aro_ (top) and PAP_val_ (bottom), predicted based on the 11 acoustic features of words**
