# Supplementary material for: Why 'piss' is ruder than 'pee'? The role of sound in affective meaning making
Source: PLoS One. 2018 Jun 6;13(6):e0198430. doi: 10.1371/journal.pone.0198430 (PMC5991420; doi:10.1371/journal.pone.0198430)
Supplement: S3 Table — (DOCX) [file pone.0198430.s004.docx]

**Summary of Fit for Ratings of Pseudowords / Valence:**

RSquare: 0.12160

RSquare Adj: 0.11268

Root Mean Square Error: 0.36266

Mean of Response: 2.79591

Number of Observations: 1095

| **Term** | **Estimate** | **Std Error** | **t Ratio** | **Prob>\|t\|** |
| --- | --- | --- | --- | --- |
| Intercept | -47.1406 | 8.07542 | -5.83754 | 6.99E-09 |
| F0 | -0.00042 | 0.002185 | -0.19442 | 0.845883 |
| F1-Mean | -0.00046 | 0.000105 | -4.34261 | 1.54E-05 |
| F2-Mean | 6.71E-05 | 8.18E-05 | 0.819816 | 0.412502 |
| F3-Mean | 3.33E-05 | 0.00012 | 0.278249 | 0.780874 |
| F1 - Bandwidth | -0.00031 | 0.000162 | -1.92997 | 0.053872 |
| F2 - Bandwidth | -0.00012 | 9.94E-05 | -1.19499 | 0.232351 |
| F3 - Bandwidth | -9.1E-05 | 9E-05 | -1.01467 | 0.310488 |
| Intensity | 0.71481 | 0.114618 | 6.236448 | 6.41E-10 |
| Intensity - SD | 0.000243 | 0.007009 | 0.034711 | 0.972316 |
| Spectral CoG | -4.7E-05 | 5.33E-05 | -0.88956 | 0.373898 |
| Spectral SD | -3.4E-06 | 3.46E-05 | -0.0994 | 0.920839 |

**S3: Summary of the regression model for the valence ratings of pseudowords (affective sound), predicted based on 11 acoustic features**
