# Supplementary material for: Why 'piss' is ruder than 'pee'? The role of sound in affective meaning making
Source: PLoS One. 2018 Jun 6;13(6):e0198430. doi: 10.1371/journal.pone.0198430 (PMC5991420; doi:10.1371/journal.pone.0198430)
Supplement: S4 Table — (DOCX) [file pone.0198430.s005.docx]

**Summary of Fit for Ratings of Pseudowords / Arousal:**

RSquare : 0.567404

RSquare Adj: 0.56301

Root Mean Square Error: 0.36168

Mean of Response: 2.95164

Number of Observations: 1095

| **Term** | **Estimate** | **Std Error** | **t Ratio** | **Prob>\|t\|** |
| --- | --- | --- | --- | --- |
| Intercept | 130.5339 | 8.053664 | 16.20802 | 4.61E-53 |
| F0 | 0.013187 | 0.002179 | 6.052399 | 1.96E-09 |
| F1-Mean | 0.000793 | 0.000105 | 7.554137 | 8.94E-14 |
| F2-Mean | 3.62E-05 | 8.16E-05 | 0.443408 | 0.65756 |
| F3-Mean | -0.00042 | 0.000119 | -3.51977 | 0.00045 |
| F1 - Bandwidth | 0.000773 | 0.000161 | 4.789613 | 1.9E-06 |
| F2 - Bandwidth | -0.00025 | 9.92E-05 | -2.56711 | 0.010388 |
| F3 - Bandwidth | 5.63E-05 | 8.98E-05 | 0.62661 | 0.531047 |
| Intensity | -1.83045 | 0.114309 | -16.0132 | 5.85E-52 |
| Intensity - SD | 0.020913 | 0.00699 | 2.991772 | 0.002836 |
| Spectral CoG | 7.81E-05 | 5.32E-05 | 1.46912 | 0.142091 |
| Spectral SD | 0.000214 | 3.45E-05 | 6.19966 | 8.03E-10 |

**S4: Summary of the regression model for the arousal ratings of pseudowords (affective sound), predicted based on 11 acoustic features**
