# Supplementary material for: Why 'piss' is ruder than 'pee'? The role of sound in affective meaning making
Source: PLoS One. 2018 Jun 6;13(6):e0198430. doi: 10.1371/journal.pone.0198430 (PMC5991420; doi:10.1371/journal.pone.0198430)
Supplement: S5 Table — (DOCX) [file pone.0198430.s006.docx]

Correlation Coefficients:

|  | **PAP_aro_** | **AS-R_aro_** | **AS-P_aro_** | **AM-R_aro_** |  | **PAP_val_** | **AS-R_val_** | **AS-P_val_** | **AM-R_val_** |
| --- | --- | --- | --- | --- | --- | --- | --- | --- | --- |
| F0 | 0.11 | 0.16 | 0.23 | 0.03 |  | -0.02 | -0.08 | -0.04 | 0.02 |
| F1-Mean | 0.42 | 0.43 | 0.78 | 0.08 |  | -0.38 | -0.40 | -0.85 | -0.06 |
| F2-Mean | 0.24 | 0.30 | 0.47 | 0.04 |  | 0.026 | -0.21 | -0.31 | 0.02 |
| F3-Mean | 0.15 | 0.08 | 0.14 | 0.03 |  | -0.03 | -0.08 | -0.19 | 0.01 |
| Intensity | -0.35 | -0.46 | -0.80 | -0.06 |  | 0.15 | 0.44 | 0.81 | 0.03 |
| Intensity - SD | 0.12 | 0.08 | 0.28 | 0.02 |  | -0.004 | -0.09 | -0.19 | 0.01 |
| F1-Bandwidth | 0.42 | 0.43 | 0.71 | 0.08 |  | -0.36 | -0.38 | -0.75 | -0.05 |
| F2-Bandwidth | 0.31 | 0.27 | 0.45 | 0.04 |  | -0.19 | -0.26 | -0.59 | -0.01 |
| F3-Bandwidth | 0.29 | 0.20 | 0.23 | 0.09 |  | -0.01 | -0.12 | -0.28 | -0.01 |
| Spectral CoG | 0.25 | 0.37 | 0.64 | 0.05 |  | -0.37 | -0.30 | -0.52 | -0.07 |
| Spectral SD | 0.29 | 0.44 | 0.70 | 0.06 |  | -0.33 | -0.36 | -0.56 | -0.06 |

**S5: Correlation Coefficients between PAP, AS-R, AS-P, AM-R and 11 acoustic variables.**

**Abbreviations**: -aro=Arousal, -val= Valence, PAP= Phonological Affective Potential, AS-R= Affective Sound based on Word Rating (study 2a), AS-P= Affective Sound Predicted based on Acoustic Features (study 2b), AM-R= Ratings of words’ Affective Meaning, i.e. original valence and arousal rating values in the database, BW= Bandwidth, SD= standard deviation
